# Developing a Multi-Dimensional Early Elementary Mathematics Screener and Diagnostic Tool: The Primary Mathematics Assessment

**DOI:** 10.1007/s10643-017-0854-x

**Published:** 2017-04-09

**Authors:** Jonathan L. Brendefur, Evelyn S. Johnson, Keith W. Thiede, Sam Strother, Herb H. Severson

**Affiliations:** 1 Department of Curriculum and Instruction, Boise State University, 1910 University Dr., MS 1725, Boise, Idaho 83725-1725 USA; 2 Department of Early and Special Education, Boise State University, Boise, USA; 3 College of Education, Boise State University, Boise, USA; 4 Initiative for Developing Mathematical Thinking, Boise State University, Boise, USA; 5 Oregon Research Institute, Eugene, USA

**Keywords:** Early childhood, Assessment screener, Mathematics

## Abstract

There is a critical need to identify primary level students experiencing difficulties in mathematics to provide immediate and targeted instruction that remediates their deficits. However, most early math screening instruments focus only on the concept of number, resulting in inadequate and incomplete information for teachers to design intervention efforts. We propose a mathematics assessment that screens and provides diagnostic information in six domains that are important to building a strong foundation in mathematics. This article describes the conceptual framework and psychometric qualities of a web-based assessment tool, the Primary Math Assessment (PMA). The PMA includes a screener to identify students at risk for poor math outcomes and a diagnostic tool to provide a more in-depth profile of children’s specific strengths and weaknesses in mathematics. The PMA allows teachers and school personnel to make better instructional decisions by providing more targeted analyses.

## Introduction

In the primary grades students are rarely tested on national and international tests, but there is ample evidence that mathematics performance at this level is not adequate (Clarke and Shinn 2004; Clements et al. 2008; Denton and West 2002; NRC 2009). Ginsburg et al. (2008) describe differences in students’ knowledge as early as age four and provide evidence of a marked difference in mathematical understanding between U.S. students and Asian students. Several researchers have also demonstrated that students who complete kindergarten with an inadequate knowledge of basic mathematics concepts and skills will continue to experience difficulties with mathematics throughout their elementary and secondary years (Duncan et al. 2007; Jordan et al. 2009; Morgan et al. 2009).

In addition, based on the poor performance of fourth grade students on national and international tests of mathematics, it is evident that students in the early grades are not adequately prepared in mathematics (Clements and Sarama 2007; Clements et al. 2008; Gersten et al. 2009; NRC 2009; Reese et al. 1997). Using large data sets and nationally representative samples, several researchers have demonstrated that students who complete kindergarten with an inadequate knowledge of basic mathematics concepts and skills will continue to experience difficulties with mathematics throughout their elementary and secondary years (Duncan et al. 2007; Jordan et al. 2009; Morgan et al. 2009). This research points to the critical need for early identification of students who are experiencing difficulties in mathematics and, then, to provide immediate and targeted intervention in order to build foundational skills and knowledge (Chernoff et al. 2007; Ginsburg et al. 2008).

There is a great need and demand for reliable, efficient, and valid primary level mathematics screening and diagnostic tools to identify students with mathematics deficiencies so teachers can intervene with differentiated lessons in order to remediate student deficiencies. Most current tools either provide inadequate diagnostic information or are too time consuming to administer on a large scale to K-2 classrooms (Brendefur et al. 2015).

## Mathematics Screeners at the Primary Level

The screening and diagnostic instruments for K-2 mathematics currently in use have not demonstrated adequate predictive validity against standardized achievement tests (Clements et al. 2008; Fuchs et al. 2007; Gersten et al. 2011). Clements and colleagues have demonstrated that most early childhood diagnostic instruments in mathematics have been limited to number concepts and do not include other important domains that are predictive of later success in mathematics. While these screeners are quick to administer, they produce an insufficient profile of student deficiencies, which results in ineffective interventions that are often misaligned to students’ needs. The current Common Core State Standards for Mathematics (CCSS-M) have placed a greater emphasis on a range of mathematical concepts, including teaching geometry and early algebra in the K-6 grades (NGA 2010). Screening for only number concepts does not evaluate other important mathematical skills necessary to succeed in these recommended standards for K-6 mathematics.

In addition, the NRC (2009) has called for better quality instruments to diagnose students’ level of competence in different areas of mathematics. The review of the extant research on mathematics skills in the primary grades supports six key areas that predict students’ later success in mathematics: concepts of sequencing, facts, relational thinking, context, measurement, and spatial reasoning. Each of these areas is critical to the development of mathematical competencies and should be evaluated in any early mathematics screener or diagnostic instrument (Clements et al. 2008; Clements and Sarama 2004).

Current comprehensive multi-item mathematics assessments such as the Test of Early Mathematics Assessment, require thirty minutes to an hour to administer (NCRTI 2011). These comprehensive assessments provide very detailed information about student needs, but are difficult to administer to a large number of young students in a timely fashion. The expenditure of resources with the longer form tests at the K-2 level is significant. A 30–45 min screener administered to a class of just 20 K-2 students will require 10–15 h to complete as opposed to a 10 min screener, which would require 3.3 h. Screening instruments have the added benefit of narrowing the size of the students at-risk pool. If general instruction is effective, most students will be found not to have any significant mathematical needs, resulting in an unnecessary use of valuable time and resources if the current, comprehensive assessments were given to all students. A multiple-gating system that uses a brief screener to quickly identify students who require further evaluation makes better use of scarce resources.

## Response to Intervention (RtI) in Mathematics

Tiered models of service delivery, such as response to intervention (RTI) models have been adopted throughout the U.S. and in an increasing number of countries (NCRTI 2011). The RtI model requires schools to analyze student learning in the context of instruction and to provide a responsive system of instruction to meet students’ needs (Gersten et al. 2009; Lyon 1995; NJCLD 2005). A major tenet of the RtI model is to conduct universal screening to identify students at increased risk for poor outcomes. Screening is often seen as the first gate of the RtI process (Mellard and Johnson 2008). The development of an efficient mathematics screener is critical for grades K-2 as early identification and intervention efforts are much more effective than waiting until the child has experienced significant difficulties.

Improving instruction is highly depending on the reliable identification of students’ mathematical needs. A screening assessment followed by a more comprehensive, targeted diagnostic assessment is an efficient and effective way of informing intervention needs. Through this process, instruction and intervention efforts are directly aligned with students’ presenting needs (Fuchs et al. 2007). More specifically, the evaluation of a student’s learning needs, coupled with intervention and progress monitoring aligned to those needs, has been shown to improve student achievement, on average, by 0.7 standard deviation units (Brophy and Good 1986; Fuchs and Fuchs 1986; Hattie 2009; Hattie and Timperley 2007; Stiggins 2005).

## Multiple-Gating Approach to Evaluation

Due to resource constraints, school districts are faced with larger class sizes and diminishing resources to serve their students. At the same time, schools face continued pressure to implement data-based decision making that results in improved student outcomes. Thus, it is necessary that the screening and diagnostic procedures be brief but accurate to maximize limited resources. Multiple-gating approaches to assessment offer a potential solution to this demand. First introduced by Cronbach and Gleser (1965), multiple-gating has been adopted for use in numerous domains in schools. The goal of multiple-gating is to quickly assess a large population, identify the potential risk pool, and then confirm and further analyze needs through a longer, diagnostic assessment administered only to those initially identified as at-risk.

Assessment resources are conserved by first administering a short universal screening procedure to all students in stage 1, which identifies students likely to be at risk (Fuchs et al. 2012). Because of limited item sampling in stage 1, students who are not at-risk may be identified. These errors are called false positives, and are common when screening young children. It is therefore important to conduct additional assessment to confirm screening results. Those students who have been identified through stage 1 screening proceed to stage 2, which includes diagnosing student needs using a more comprehensive diagnostic tool.

The stage 2 evaluation serves two basic functions: first, it creates a more complete picture of each student’s deficiencies, providing more diagnostic information for devising a remediation program; second, it helps identify false positives from the stage 1 screening procedure, thus reducing the unnecessary expenditures of further resources. Multiple-gating can substantially improve the quality of educational decision-making. When implemented with integrity, it is a highly accurate and cost effective procedure (Walker et al. 2014). Multiple-gating systems have been shown to reduce resource consumption by as much as 58% as opposed to the use of single stage screening procedures (Loeber et al. 1984).

**Fig. 1:**
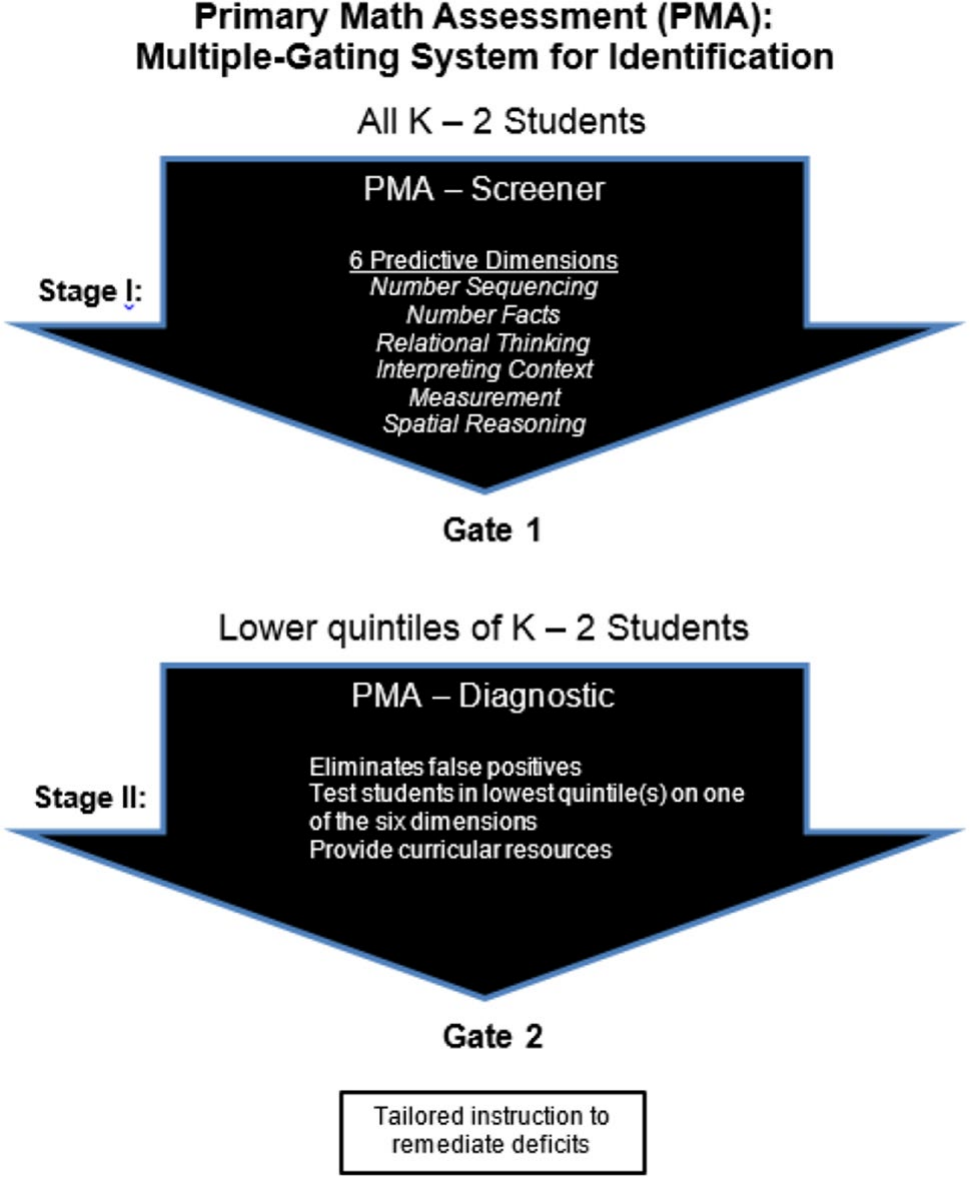
Primary-Mathematics Assessment (PMA) multiple-gating diagram

To address these issues within the area of early mathematics instruction, we created a two stage evaluation system for the early identification of students at-risk for poor math outcomes (see Fig. 1) (Brendefur et al. 2015).

## The Primary Math Assessment (PMA)

**Fig. 2:**
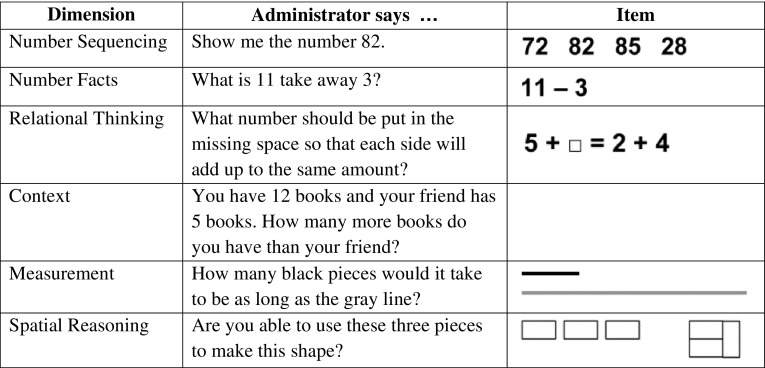
PMA-S sample items

The PMA consists of the PMA-Screener (PMA-S) and the PMA-Diagnostic (PMA-D). The PMA-S is used to quickly identify students at-risk for poor math outcomes. It is a research-validated, universal screening tool that assesses domains beyond simple number concepts (Brendefur et al. 2011) and has been administered to over 10,000 students per grade level over the past 3 years. The conceptual framework incorporates concepts of number, relationships, context, measurement, and spatial reasoning. Sample items for each domain are displayed in Fig. 2. The concepts evaluated by the PMA-S provide a more complete picture of student strengths and deficiencies than existing screeners. The PMA-S takes approximately 8 min to administer.

The PMA is administered and scored online. The web version provides teachers with immediate feedback on student performance, making it possible to quickly identify students at-risk for poor math outcomes. For each class, the web version creates reports, identifying students with similar needs to assist the teacher in creating homogenous groups for remediation instruction. Also, the web version allows for individual student results to be compared with a class, school district or state-wide norms.

**Fig. 3:**
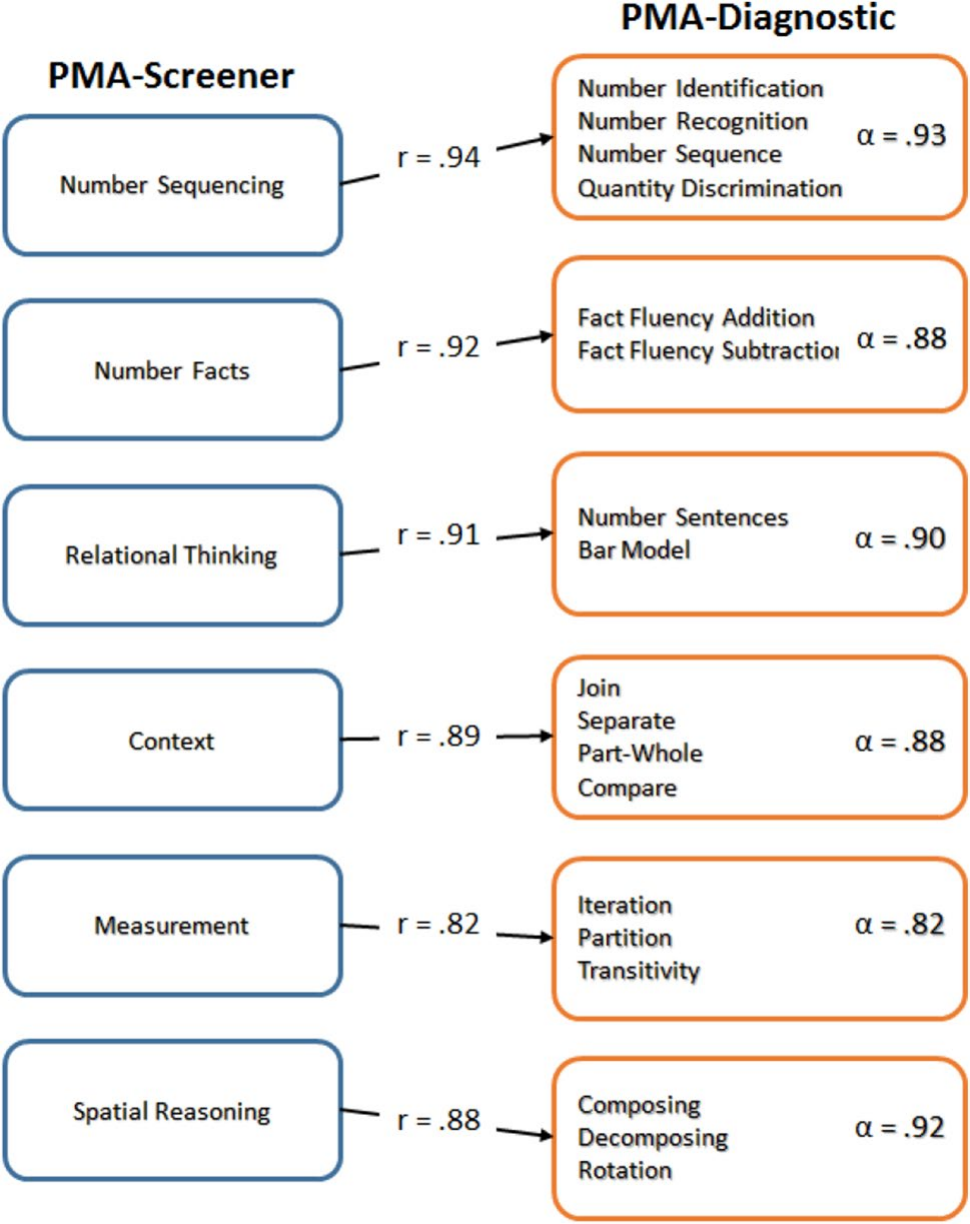
The PMA-S and PMA-D relationships

Students who are identified as at-risk on the PMA-S are tested with the PMA-D, a diagnostic test that includes 64 items. For each of the six dimensions, students can be tested on any or all of these more targeted assessments to determine more specific areas of need. Based on the results of the PMA-S, a student can be tested on the specific PMA-D area to determine more specific deficiencies related to sub-dimensions within measurement (see Fig. 3).

To examine the psychometric properties of the diagnostic tool, the PMA-D was administered to over 500 K-2 students. Each dimension has a strong internal consistency and is highly correlated to the screener items. Number sequencing has an internal consistency of 0.93 and consists of four sub-constructs: number identification, number recognition, number sequencing, and quantity discrimination. Number facts has an internal consistency of 0.88 and consists of fact fluency items for addition and subtraction problems. Relational thinking has an internal consistency of 0.90 and includes number sentences with missing values and bar model situations. Context has an internal consistency of 0.88 and consists of join, separate, part-whole, and compare problem types. Measurement has an internal consistence of 0.82 and consists of iteration, partition, and transitivity items. The final sub-dimension is spatial reasoning with an internal consistency of 0.92. The spatial reasoning items consist of composing, decomposing, and rotation of two-dimensional geometric figures. The overall reliability of the PMA-D for all three grades reported Cronbach’s alphas between 0.82 and 0.93, which is considered to be high (DeVellis 1991; Nunnally 1978).

## Conclusion

Thiede and colleagues (Thiede et al. 2012) have demonstrated that teachers are often not able to determine which students are at-risk for poor learning outcomes and require additional support. The PMA system has the ability to create homogenous groups of students who can be grouped by their specific mathematical learning needs to aid the teacher in providing targeted group instruction. In an environment of increasing demands and class size, the ability to produce immediate test results, homogenous grouping by need and curricular recommendations will support teachers in providing more efficient and effective intervention to support the needs of their students. The PMA has the potential to help result in more efficient teaching and improved educational outcomes.
